# Association of Oral Status and Early Primary Hypertension Biomarkers among Children and Adolescents

**DOI:** 10.3390/ijerph17217981

**Published:** 2020-10-30

**Authors:** Elzbieta Paszynska, Monika Dmitrzak-Weglarz, Danuta Ostalska-Nowicka, Michal Nowicki, Maria Gawriolek, Jacek Zachwieja

**Affiliations:** 1 Department of Integrated Dentistry, Poznan University of Medical Sciences, 60-812 Poznan, Poland; mgawriolek@gmail.com; 2 Psychiatric Genetics Unit, Department of Psychiatry, Poznan University of Medical Sciences, 60-806 Poznan, Poland; mweglarz@ump.edu.pl; 3 Department of Pediatric Nephrology and Hypertension, Poznan University of Medical Sciences, 60-572 Poznan, Poland; dostalska@ump.edu.pl (D.O.-N.); jacekzachwieja@ump.edu.pl (J.Z.); 4 Department of Histology and Embryology, Poznan University of Medical Sciences, 60-781 Poznan, Poland; mnowicki@ump.edu.pl

**Keywords:** caries, oral health, hypertension, uric acid, creatinine, cystatin C

## Abstract

The aim of this case–control study was the evaluation of the association between biomarkers of early primary arterial hypertension (HA) and oral diseases among children and adolescents. Material and methods. Subjects suspected of primary HA (n = 180) underwent a complex evaluation of their vascular status: blood pressure, heart rate, vascular stiffness, sympathetic activity in a 24 h ambulatory examination, followed by measurement of serum uric acid (UA), cystatin C, and creatinine. This procedure allowed the identification of children with primary (n = 58) and secondary HA (n = 74), as well as of children with normal arterial blood pressure, who served as a control group (n = 48). All subjects with secondary HA were excluded from further investigation. Oral examination included the measurement of caries intensity (using the decayed, missing, filled index for permanent teeth DMFT /primary teeth dmft), bacterial plaque (by the plaque control record index, PCR%), and gingivitis (by the bleeding on probing index, BOP%). For statistical analysis, a linear regression model and Spearman rank correlation were used. Results. UA, cystatin C, and creatinine were not altered in the HA group. However, the number of decayed permanent teeth (DT) and the DMFT, PCR%, and BOP% indexes were significantly higher in the primary HA group compared to the control group (*p* = 0.0006; *p* = 0.02; *p* = 0.0009; *p* = 0.003). Our results are not sufficient to prove the important role of caries and gingival inflammation in the modulation of HA symptoms, although they prove the association of oral diseases with primary HA symptoms. This may indicate future strategies for preventive measures for hypertensive children and adolescents.

## 1. Introduction

Unbalanced and dysbiotic oral bacteria have been proposed to play a role in a number of systemic diseases like hypertension, allergy, cancer, cardiovascular disturbances, diabetes, rheumatoid arthritis, and others [1,2,3]. It is possible that a poor oral status may also influence the induction of primary hypertension among children [4,5,6,7], although the association of primary arterial hypertension (HA) with tooth decay has still not been convincingly described. Primary HA in children and adolescents is an increasingly common issue in medicine [8]. It usually persists in adulthood and may lead to life-threatening complications [9].

The etiology of primary HA is multifactorial. In childhood, there are several risk factors for HA, e.g., preterm delivery, overgrowth/obesity, hyperuricemia, which may cause subclinical inflammation that may influence endothelial activity [10,11,12,13]. The independent association of HA with hyperuricemia has been analyzed in multiple studies [13,14,15]. Several markers are evaluated to confirm systemic hyperuricemia and renal deviations: high serum uric acid (UA), cystatin C, and creatinine. However, hyperuricemia, high serum UA, cystatin C, and creatinine concentrations cannot lead to pathological effects, such as oxidative stress and inflammation. For this reason, hyperuricemia is considered an independent risk factor for the permanent remodeling of the vascular bed due to endothelial cells damage and muscular hyperplasia [14,15]. Interestingly, often there are no additional clinical symptoms. Cystatin C is mainly used as a biomarker of kidney function and recently has been studied for its role in cardiovascular diseases [16,17,18]. Therefore, it seemed logic to include cystatin C in our HA examination.

The aim of the current case–control study was the evaluation of the association between biomarkers of early primary HA with oral diseases (dental caries, gingival inflammation) among children and adolescents.

## 2. Materials and Methods

### 2.1. Participants and Procedures

This case–control study was carried out at the Poznan University of Medical Sciences. The study followed the rules of the Declaration of Helsinki and complies with Good Clinical Practice guidelines. Approval was obtained from the Bioethics Committee at the Poznan University of Medical Sciences (resolution No. 1166/19). The course of the study was explained to all participants, who provided their written informed consent to participate in the study.

A clinical trial was conducted among all children admitted to hospital with suspected primary HA. One hundred and eighty patients were involved in the present study between January 2019 and January 2020. A hospital protocol was followed for the diagnosis of primary HA as a complex evaluation of the vascular status at the Department of Pediatric Nephrology and Hypertension, Poznan University of Medical Sciences, funded by a Polish National Health Fund [19]. A reason of hospitalization was the verification of arterial hypertension and its final confirmation. Medical and dental assessments were carried out in the same manner in a blind fashion for each child/adolescent, before the final diagnosis, independently of whether the child was affected by primary arterial hypertension or other disease or was healthy. Therefore, all the children were hospitalized to verify their medical history, perform 24 h arterial blood pressure monitoring (ABPM) [20], echocardiography (ECHO), electrocardiography (to exclude masked hypertension), and laboratory tests, i.e., analysis of peripheral blood morphology white blood cells WBC, red blood cells RBC, blood plates PLT, hemoglobin Hb, hematocrit HCT, of the levels of creatinine, cystatin C, uric acid, urea UA, Na, K, Ca, Mg, total cholesterol, low density lipoprotein LDL-cholesterol, glucose, thyreotropic stimulating hormone TSH, triiodothyronine fT3, free fraction of thyroxine fT4, liver enzymes, total protein, C-reactive protein CRP, and of the 24 h urine levels of protein and albumin [20,21].

All laboratory tests served to exclude comorbidities. Diagnosis of HA was crucial to divide the individuals into study or control groups and to exclude subjects from the study due to additional diseases.

Seventy-four subjects were not admitted to the study, as they satisfied at least one of the following exclusion criteria: (1) age <6 years, (2) secondary hypertension (resulting primarily from reflux nephropathy, polycystic kidney diseases, unilateral renal agenesis, and renal dysplasia, (3) heart failure, (4) hepatic dysfunction, (5) autoimmune diseases, (6) diabetes, (7) coexistence of neoplastic and/or any infectious diseases, (8) history of oral contraceptive use, or (9) current therapy with the use of medications known to affect serum uric acid levels [20,21].

The protocol presented above allowed the identification of 58 children with primary HA (HA group, n = 58) and 48 children who were allocated to the control group (Ctrl group, n = 48). Besides medical examination and evaluation according to the inclusion/exclusion criteria protocol, children were matched according to ethnic group and geographical region; the schedule of vaccination was maintained.

The control children were considered normotensive based on careful verification of their medical history, ABPM, and laboratory values.

Subjects enrolled into the control group had to meet the following inclusion criteria: (1) age 6–18 years, (2) no arterial hypertension, (3) normal clinical examination without a history of chronic diseases, (4) no clinical or laboratory signs of infection, (5) normal concentration of serum cortisol, glucose, thyroid hormones, electrolytes, (6) no aberrations in ECHO and ECG, (7) no complications in the course of the perinatal period, (8) signed informed consent [20,21].

No study participant was taking any anti-hypertensive or other medications such as anti-inflammatory drugs.

With our sample size, this study had 75% power for comparison of means in two independent samples in the hypertensive children group and 85% power in the control children group, at the *p* < 0.05 [22,23].

#### 2.1.1. Biochemical Analysis

Vascular examination was followed by the collection of peripheral blood samples (during the morning) [24]. Venous blood was collected upon morning admission (8–9 am) after overnight fasting. Serum was immediately separated from the blood by centrifugation at 1000 x g for 15 min at 4 °C, aliquoted into Eppendorf tubes, frozen at −70 °C, and assayed afterward. Laboratory measurements of uric acid, cystatin C, creatinine were performed using a standardized method specific for these components, according to the manufacturer’s instruction.

#### 2.1.2. Dental Examination

During the 24 h vascular measurement, dental examination was carried out at the dental office. The clinical dental examination included the evaluation of dental tissues, oral hygiene, and gingival inflammation using standardized indicators (see description below). Scoring of the occlusal, buccal, and lingual teeth surfaces was performed after their cleaning and drying (excluding the third molars) under good lighting, without magnification. Prior to the study, the examiner was trained (E.P.). Dental examination records included the number of decayed teeth, the number of restored teeth by fillings, and the number of missed teeth, both milk and permanent teeth, using the decayed, missing, filled teeth (DMFT/dmft, capital letters for permanent teeth, lower case letter for primary teeth) score, evaluating dental caries [25].

Dental plaque and gingival status were recorded by using a manual graded periodontal probe (LM-instruments, LM8 5050 probe, Osakeyhtiö, Parainen, Finland). Plaque control was evaluated using the dichotomized plaque control record index (PCR), [26]. Gingival inflammation was determined using the bleeding on probing index (BOP), [27], measured in six points of the gingival sulcus of all teeth (excluding the third molars). The proportion of surfaces (%) with dental plaque or bleeding-on-probing gums, respectively, were calculated for each individual child as % of sites [28].

The oral part of the investigation was based on a clinical examination performed by one examiner (E.P.), prior to the final medical diagnosis and division of the subjects into groups. It was important that all the children if the control group (Ctrl) were examined blindly before their final assignment to the healthy or the unhealthy group.

The examiner involved in the oral assessment was trained before the onset of the survey under the guidance of an experienced dentist. The dentist was trained at the start of the study and retrained twice more during the course of the study, using a training tool specially developed for this study, comprising the assessment of almost 30 clinical photographs of teeth with different scores in a random sequence. To test inter-examiner reliability, linear (0.82, 0.84, 0.83) and squared (0.92, 0.94, 0.93) weighted Kappa coefficients were calculated Table S1.

### 2.2. Statistical Analysis

The Lillefors and Shapiro–Wilk tests were used to test the normality of the data. The homogeneity of variance of each variable was calculated with the Levene’s test. Non-parametric tests were applied in the analyses of data with non-normal distribution. Because most of the variables presented a non-normal distribution and the homogeneity of variance was violated, we used a non-parametric Mann–Whitney U-test. Correlation with clinical and biochemical parameters was performed using Spearman’s correlation rank test. A linear regression model was employed to evaluate dependent categorical variables (“hypertensive children HA “ vs. “control patients Ctrl”) with qualitative and quantitative predictors (body mass index (“BMI”), “serum uric acid concentration”, “serum creatinine concentration”, “serum cystatin C concentration”, “PCR%”, “BOP%”, decayed permanent teeth (“DT”), decayed primary teeth (“dt”), caries score for secondary teeth “DMFT”, caries score for primary teeth “dmft”). The statistical significance level was set at *p* < 0.05. Statistical analyses were conducted using the Statistica v12 software (StatSoft Inc., Tulsa, USA).

## 3. Results

### 3.1. Demographic and Clinical Characteristics

Sample: The final sample consisted of 106 participants (58 HA and 48 control subjects). The mean age of the HA patients was 14.3 ± 2.7 years, the mean age of the controls was 13.2 ± 3.4 years, with no statistically significant difference (*p* = 0.094). Body weight was statistically different between patients and controls (*p* < 0.001), as their BMI (*p* < 0.0004). The demographic characteristics of all participants are presented in Table 1.

Uric acid, cystatin C, creatinine concentrations. Median levels in the blood were not significantly different between HA and Ctrl groups (*p* = 0.065; *p* = 0.216; *p* = 0.135), (Table 1).

#### 3.1.1. Dental Examination

Analysis of the oral health revealed that HA children presented a significant higher number of DT, (*p* < 0.0006) and higher DMFT score (*p* < 0.021), amount of dental plaque (PCR%), (*p* < 0.0009), and gingival inflammation as BOP% index, (*p* < 0.003), (Table 1). However, no differences in the results of dental examination of the primary dentition were detected between HA and healthy individuals of the Ctrl group (*p* > 0.05).

#### 3.1.2. Correlations between the Analyzed Variables

In the HA group, Spearman analysis evidenced a significant correlation between uric acid levels and creatinine levels, BMI, dt, dmft, between cystatin C and DT, PCR%, and between creatinine levels and cystatin C levels, BMI, DMFT, dmft, dt, PCR% (Table 2).

In the Ctrl group, statistical analysis found significant correlation between uric acid and creatinine, cystatin C, BMI, cystatin C, and between BMI, creatinine and BMI, dt, dmft (Table 2).

#### 3.1.3. Linear Regression Model

For a few cases, we obtained significance for the tested pairs; however, most cases presented a weak linear relationship, in both (HA/Ctrl) groups, for UA, creatinine, and BMI, UA and creatinine, dt, dmft, and creatinine. Therefore, for such variables, we analyzed the correlation by measuring the strength of a relationship that does not have to be linear (see Supplementary Material Table S2).

## 4. Discussion

Previous studies in a HA population, which could help interpreting our results, are lacking. We propose that HA induction and worse parameters for the HA group could be explained by a prolonged oral bacteria stimulation and inflammatory burden. In the study group, we found significantly higher values of indicators referring to gingival inflammation (PCR, BOP). Other outcomes based on clinical cross-sectional studies and meta-analyses indicate the association between chronic periodontitis and hypertension, without any evidence that a periodontitis therapy could reduce hypertension [29]. Current epidemiological data show a link between hypertension and periodontitis; however, there is no strong evidence that a causal relationship exists [30]. Additional issues need to be addressed to improve the overall management of patients’ health [30]. Therefore, a new, contemporary definition of caries based on changes in microbiome activity could be useful. It might confirm our previous observation that the only factor correlated with hypertension in the study group was tooth decay. Other clinical or laboratory parameters, due to their secondary nature, seem to have a lower predictive value. Nevertheless, it seems that the maintenance of caries-free teeth in the oral cavity is important for oral and vascular health reasons [31]. An atherosclerotic plaque molecular analysis at variable activity found the presence of *Streptococcus mutans* (SM), *Prevotella intermedia* (PI), *Porphyromonas gingivalis* (PG), *Treponema denticola* (TD), with the highest prevalence of SM [31]. In HA patients with poor oral status, we also observed intensive oxidation of several substrates [32], whose consequence might be an increase in the levels of reactive metabolites of oxygen, lipid peroxidation, total antioxidant capacity. This could result in the inactivation of prostacyclin and NO, hence, an enhancement of peripheral vascular resistance and hypertension [33]. In our study, the oral status of the HA group was undoubtedly uncorrected due to caries and poor oral hygiene. Therefore, our clinical implications suggest that HA patients at a young age need prevention measures regarding their oral care and hygiene. They should be followed up in shorter intervals between dental examinations, because it has previously been demonstrated that the risk of new caries, as well as the progression of lesions already present, is the highest during early adolescence [34,35].

The most interesting finding of the present study is that UA concentrations remained within ranges generally considered physiological [36]. However, in our opinion, this phenomenon should be further discussed. As it was already mentioned, UA is systematically released from damaged cells to form urate crystals, which are recognized by immune system cells that secrete interleukin-1 [37]. This typical proinflammatory cytokine initiates an endovascular inflammatory process leading primarily to endothelial failure, a process that finally may lead to atherosclerosis. Chronic deciduous tooth decay, dental plaque, and gingival bleeding in children and adolescents can be then local triggering signals affecting UA concentration and the initiation of the inflammatory cascade in the endothelium, of which HA is the first clinical manifestation [38,39].

A significant correlation of hyperuricemia with primary HA has been reported in numerous multicenter studies and clinical trials [13,14,15]. First of all, these trials describe the benefits of allopurinol use (a xanthine oxidase inhibitor), not only leading to blood pressure normalization, but also reversing endothelial cell dysfunctions [40,41]. In line with the above, we postulate that even a small increase in serum UA concentration in HA patients, not exceeding the upper level of the normal values, should be treated as an indicator of actively ongoing inflammation in the vascular bed.

Similarly to UA, cystatin C concentrations might indicate the beginning of glomerulopathy, although they were within normal limits [42]. However, glomerular filtration rate GFR is the most important parameter used to define renal function in primary and secondary urinary system diseases. GFR is routinely calculated using serum creatinine concentration and body surface area (BSA)-based formulae [18]. However, these calculations may give incorrect results, particularly for obese children, due to a higher BSA than in normal-weight children. Cystatin C is a known kidney function marker that reflects the correct GFR [16,17]. Certainly, the changes described above were still subclinical because they were not accompanied by differences in creatinine concentration, GFR values, or urinalysis. However, as it was reported for the UA analysis, in HA children and adolescents with developed carious lesions, cystatin C values, already at the upper level of the normal values, should be considered an early indicator of nephropathy [43,44].

The significant BMI difference between primary HA patients and controls needs a separate discussion. Generally, obesity is considered a pathological condition due to an increased amount of adipose tissue, leading to impairments in the human body and to an increased risk of morbidity and mortality. In many reports, BMI is considered an indicator of excess weight rather than of excess fat [45]. For this reason, some other factors like the BMI z score and a “body shape index” have been introduced into clinical practice to measure the effect of visceral fat on the development of HA and atherosclerosis. In our previous report, however, we did not show significant differences in BMI, BMI z score, and “body shape index” [45]. All three factors, in children and adolescents, were characterized by the same sensitivity and specificity. For this reason, in the present report, we focused only on BMI. It is well known that increased BMI correlates with increased expression of various proinflammatory cytokines activating the vegetative and central nervous systems (CNS) [46,47]. In our study group, there were only a single patient with obesity and three overweight patients. There are no extensive reports in the literature about the direct relationship between obesity and tooth decay. Perhaps, this relationship is due to improper eating habits, which can also lead to chronic inflammation in the mouth, but further research on caries in pediatric groups is necessary [48,49].

### 4.1. Strengths of the Study

First, considering the paucity of data related to the topic, our study provides interesting insights about the association of oral disease with HA symptoms, despite the small number of subjects.

The oral part of the investigation was based on clinical examinations performed by one examiner in a dental office and, importantly, prior to the final medical diagnoses. Especially, the last criterion excluded any additional bias in dental plaque or gingival evaluations. According to subjective circumstances, all children were examined blindly before their final separation into healthy or unhealthy groups.

### 4.2. Limitations of the Study

No previous information related to pre-diagnostic values (baseline) was available for our clinical samples. In addition, it would be interesting to extend the measurements to longer period of time, including monitoring the HA patients after full oral care. The number of participants who was finally included (over 100 subjects) is small, and the research has to be extended to larger pediatric groups. Moreover, our investigation was also limited by its case–control design and by the evaluation of clinical measures of the oral status based on visual criteria during oral assessment. The use of digital instruments for caries detection seems to be more accurate, but other difficulties limit it. One of the possible confounders was also the lack of parental examination and the hereditary burden. Parents and family members were not included in the hospital examination, only a general interview was conducted. All mentioned weaknesses do not, however, undermine the representativeness of the groups we examined.

## 5. Conclusions

Although uric acid, cystatin C, and creatinine concentrations were still within normal limits, these indicators of HA nephropathy correlated with tooth decay and gingival inflammation. Even if our results are not enough to prove the important role of caries and gingival inflammation in the modulation of HA symptoms, we proved the association of oral diseases with HA symptoms. Therefore, the evaluation of the oral status may indicate future strategies for caries prevention among hypertensive children and adolescents.

## Figures and Tables

**Table 1:** Anthropometric, biochemical, and oral data, for HA and Ctrl individuals.

Variables	HA (n = 55) Mean ± SD Median (min-max)	Ctrl (n = 48) Mean ± SD Median (min-max)	*p*-Value
age [years]	**14.3** ± 2.7 ** 15 (7–18)	**13.2** ± 3.4 14 (4–18)	ns
uric acid	**5.4** ± 1.5 5 (2–9)	**4.9** ± 1.2 5 (3–8)	0.065
cystatin C	**0.9** ± 0.3 1 (0–3)	**0.8** ± 0.1 1 (1–1)	ns
creatinine	**0.6** ± 0.2 1 (0–1)	**0.6** ± 0.2 1 (0–1)	ns
BMI [kg/m^2^]	**26.2** ± 6.8 25 (14–43)	**21.9** ± 5.0 21 (14–32)	0.0004
DT	**2.0** ± 3.8 1 (0–22)	**0.3** ± 0.6 0 (0–2)	0.0006
MT	**0.2** ± 0.8 0 (0–6)	**0.0** ± 0.1 0 (0–1)	ns
FT	**1.8** ± 2.8 1 (0–15)	**1.5** ±2.4 0 (0–10)	ns
DMFT	**3.9** ± 5.1 3 (0–28)	**1.9** ± 2.5 1 (0–1)0	0.02
dt	**0.2** ± 1.2 0 (0–9)	**0.2** ± 0.6 0 (0–3)	ns
mt	**0.0** ± 0.1 0 (0–1)	**0.0** ± 0.2 0 (0–1)	ns
ft	**0.1** ± 0.3 0 (0–2)	**0.5** ± 1.1 0 (0–5)	ns
dmft	0.3 ± 1.3 0 (0–9)	0.7 ± 1.5 0 (0–7)	ns
DMFT + dmft	**4.3** ± 5.2 3 (0–28)	**2.1** ± 2.6 1 (0–10)	0.028
Number of primary teeth	**26.1** ± 4.8 28 (10–32)	**23.5** ± 7.1 28 (0–30)	ns
Number of secondary teeth	**1.2** ± 3.2 0 (0–12)	**2.9** ± 5.0 0 (0–20)	ns
PCR%	**44.8** ± 32.6 35 (5–100)	25.5 ± 21.2 17(2–80)	0.0009
BOP%	**34.6** ± 33.9 20 (0–100)	**15.8** ± 15.9 9 (0–50)	0.003

Results are expressed as **mean** ± standard deviation, ** Median (min–max ranges). Statistical significance is given according to *p*-value (*p* ≤ 0.05, *p* ≤ 0.01, *p* ≤ 0.001) vs. non-significance (ns). Abbreviations: n, number of patients; HA, patients diagnosed with primary hypertension; Ctrl, control group, healthy children. Statistical tests used were Mann–Whitney U test, *t*-test, and Welch test. Abbreviations: BMI, body mass index; DT, number of decayed secondary teeth; MT, number of missing secondary teeth; FT, number of filled secondary teeth; DMFT, decayed, missing, filled teeth score, evaluating dental caries in permanent teeth; dt, number of decayed primary teeth; mt, number of missing primary teeth; ft, number of filled primary teeth; dmft score, total score evaluating the number of decayed, missing, filled primary teeth; PCR%, plaque control record index; BOP%, bleeding on probe index.

**Table 2:** Significant results of the Spearman’s correlation rank test regarding clinical and biochemical parameters (*p* < 0.05) for the HA and Ctrl groups.

HA Group n = 58	Spearman R	*p*-Value	Ctrl Group n = 48	Spearman R	*p*-Value
UA and creatinine UA and BMI UA and dt UA and dmft	0.46 0.565 −0.366 −0.369	0.0004 0.0000007 0.006 0.006	UA and creatinine UA and cystatin C UA and BMI	0.351 0.509 0.591	0.02 0.002 0.00003
cystatin C and DT cystatin C and PCR%	0.278 0.362	0.046 0.008	cystatin C and BMI	0.447	0.009
creatinine and cystatin C creatinine and BMI creatinine and DMFT creatinine and dt creatinine and dmft creatinine and PCR%	0.428 0.377 0.304 0.347 0.415 0.293	0.001 0.005 0.02 0.009 0.002 0.02	creatinine and BMI creatinine and dt creatinine and dmft	0.425 −0.435 −0.586	0.003 0.003 0.00001


Abbreviations: UA, uric acid.

## Supplementary Materials

The following are available online at https://www.mdpi.com/1660-4601/17/21/7981/s1, Table S1 (Supplementary): Overview of linear and squared weighted Kappa coefficients as measures of interrater reliability (re-training 1 and 2 was performed during the course of the study). Table S2 (Supplementary): Significant results of linear regression test with clinical and biochemical parameters (*p* < 0.05) between HA and Ctrl groups.
