# Supplementary material for: Association of Oral Status and Early Primary Hypertension Biomarkers among Children and Adolescents
Source: Int J Environ Res Public Health. 2020 Oct 30;17(21):7981. doi: 10.3390/ijerph17217981 (PMC7662220; doi:10.3390/ijerph17217981)
Supplement: Supplementary file 1 [file ijerph-17-07981-s001.pdf]

Table 1A (Supplementary). Overview of linear and squared weighted Kappa coefficients as measures of interrater reliability (re-training 1 and 2 was performed during the course of the study).

No. of training	Mean of linear weighted Kappa coefficients	Mean of square weighted Kappa coefficients
1 (Initial training)	0.82	0.91
2 (retraining 1)	0.84	0.93
3 (retraining 2)	0.83	0.92

Table 3 (Supplementary). Significant results of linear regression test with clinical and biochemical parameters ($p<0.05$) between HA and Ctrl groups.

**HA group n=58**	**R2**	**p-value**	**Ctrl group n=48**	**R2**	**p-value**
UA & creatinine	0.1	0.04	UA & creatinine	0.1	0.02
UA & BMI	0.3	0.00004	UA & cystatin C	0.01	0.4
UA & dt	0.00009	0.1	UA & BMI	0.3	0.000005
UA & dmft	0.04	0.2			
cystatin C & DT	0.007	0.7	cystatin C & BMI	0.006	0.6
cystatin C & PCR%	0.04	0.3			
creatinine & cystatin C	0.05	0.2	creatinine & BMI	0.08	0.03
creatinine & BMI	0.2	0.008	creatinine & dt	0.07	0.04
creatinine & DMFT	0.03	0.2	creatinine & dmft	0.1	0.01
creatinine & dt	0.2	0.007			
creatinine & dmft	0.2	0.0006			
creatinine & PCR%	0.003	0.7			

Abbreviations: n= number of patients; HA=patients diagnosed with primary hypertension; Ctrl= control group, healthy children; BMI= Body Mass Index [kg/m^2]; DMFT score= decayed missing filled secondary teeth; DT= number of decayed permanent teeth; UA- uric acid; dt= number of decayed primary teeth; dmft score= decayed missing filled primary teeth; PCR%= plaque control record index; BOP%= bleeding on probe index.
